# Knowledge, attitude, and diabetes self-care among individuals at high-risk of diabetes-related blindness in Bangladesh: a cross-sectional study

**DOI:** 10.1186/s12889-024-20772-7

**Published:** 2024-11-25

**Authors:** Shahina Pardhan, Md. Saiful Islam, Raju Sapkota

**Affiliations:** 1 Vision and Eye Research Institute, School of Medicine, Anglia Ruskin University, East Road, Cambridge, UK; 2 Centre for Inclusive Community Eye Health, School of Medicine, Anglia Ruskin University, East Road, Cambridge, UK; 3 Department of Public Health and Informatics, Jahangirnagar University, Savar, Dhaka, 1342 Bangladesh

**Keywords:** Attitude, Bangladesh, Complications, Diabetes, Duration of diabetes, Insulin treatment, Knowledge, Practice

## Abstract

**Background/Aim:**

Adequate knowledge, attitude, and self-care practice (KAP) are paramount in reducing diabetes complications. This study examined diabetes-related KAP in individuals who have been previously reported to be at a higher risk of blindness such as those on insulin treatment or with a longer (>6 years) duration of diabetes in Bangladesh.

**Methods:**

Six hundred community-dwelling individuals (mean age = 52.7±11.6 years) who had been diagnosed with diabetes by their doctor were interviewed. A semi-structured questionnaire obtained self-reported information about diabetes-related KAP, duration, treatment of diabetes, and sociodemographic parameters including age, gender, and education level. Data were collected using a purposive sample technique and analyzed using Fischer’s exact test or independent samples t-tests.

**Results:**

There were 271 males (45.2%) and 329 (54.8%) females. Of the total participants (mean diabetes duration = 6.6±6.2 years), 36.5% had diabetes for more than the median duration of 6 years, 80.7% were receiving insulin or insulin combined with tablets (*insulin* group) and the remaining 19.3% were on tablet only and/or diet control (*non-insulin* group). One-fifth (19.8%) of all the participants did not consider diabetes a serious disease, 31.3% were unaware that uncontrolled diabetes can cause blindness, 40.5% had never had their eyes tested for diabetic retinopathy and 41.5% stated that they would not attend diabetic retinopathy screening until their eyesight became worse. Among those in the insulin group, 42.1% reported being unaware that smoking may be harmful to diabetes compared to 30.2% of those in the non-insulin group (*p*= 0.02). Additionally, 64.7% of those in the insulin group were unaware that a diabetic retinal screening is different from a routine eye test for spectacles, compared to 44.8% in the non-insulin group (*p*< 0.001). Sixty-two percent of participants with diabetes duration of more than 6 years reported that diabetes management was a shared responsibility between the doctor and the patient compared to 48.3% with a shorter duration (*p*< 0.001). Those with a longer duration of diabetes (>6 years) also reported forgetting to take their medication more often than those with a shorter duration (*p* = 0.02). Twenty-one percent of participants with a duration of diabetes longer than six years had checked their eyes within the previous year compared to 63.5% of those with a shorter duration of diabetes (*p*< 0.001).

**Conclusion:**

Individuals on insulin treatment demonstrated poorer knowledge and awareness of diabetes and diabetes eye screening. Those with a longer diabetes duration exhibited poorer self-care practices, particularly not taking the medication regularly, and neglecting diabetic retinal checkups. These issues need to be addressed in designing targeted educational interventions to prevent blindness from uncontrolled diabetes in the high-risk groups in Bangladesh.

## Introduction

Diabetes mellitus is a rapidly growing public health problem, with more than 540 million people estimated to be affected by it worldwide [1, 2]. The global age-standardized prevalence of diabetes is reported to be 6·1% [3]. According to the International Diabetes Federation Atlas (tenth edition, 2021), approximately 10.5% of the global adult population aged between 20 and 79 years had diabetes in 2021, with almost half of them being unaware that they were living with the condition [2].

Bangladesh ranks eighth among countries with the highest number of diabetes [2], with approximately 13.1 million (12.5%) adult cases reported in 2021 [4]. This number is projected to double by the year 2045 [2]. A higher prevalence of diabetes has been reported among people living in urban areas compared to those in rural areas of Bangladesh [5, 6].

Several factors are known to explain why diabetes is highly prevalent in Bangladeshi and other South Asian communities. These include, but are not limited to, biological components (genetic predisposition, increased visceral fat) [7–9], rapid urbanization [10], sedentary lifestyle [11, 12], unhealthy dietary habits [13, 14], and decreased health awareness [15, 16].

Improved knowledge and awareness about diabetes are vital for promoting its self-care practices and in reducing retinopathy complication of uncontrolled diabetes [17]. A growing body of literature from Bangladesh reported a significant lack of knowledge about the causes and management of diabetes, including the risk of diabetic retinopathy in both the general and diabetic population [18–20]. Further evidence showed that people’s knowledge and understanding of diabetes are significantly associated with age, gender, education level, and family income [21]. The lack of adequate health awareness campaigns and the health system’s greater focus on managing infectious diseases rather than non-infectious diseases like diabetes are also believed to contribute to the lack of knowledge and awareness about diabetes and its control among the general public in Bangladesh [22, 23].

In addition to improved knowledge and awareness about diabetes, fostering a positive attitude towards disease management and promoting behaviors such as eating a balanced diet, exercising, and refraining from tobacco and alcohol consumption are also vital for enhancing self-care practices of diabetes control and reducing the complications of uncontrolled diabetes [24–26]. A cross-sectional study conducted in the rural communities of the Faridpur district in Bangladesh among people aged 30 years or older reported that as high as 45% of participants were unaware of any symptoms of diabetes, only 33% knew any causes of diabetes, and 14% had ever taken blood glucose tests for diabetes [22]. Additionally, 78% of people who self-reported having diabetes said they monitored their blood glucose levels less frequently than at least once a month [22]. Another study conducted in a hospital setting in Dhaka showed that a very high proportion (87%) of participants presented with poorly controlled diabetes, with fasting blood glucose levels found to be ≥7.2 mmol/L [26]. These findings highlight a pressing need for campaigns and interventions to promote awareness and self-care practices of diabetes control in both the diabetic and general population in Bangladesh.

People on insulin treatment or with a longer duration of diabetes have been shown to be at higher risk of developing diabetic retinopathy from uncontrolled diabetes [27]. A study from Nepal [16], a neighboring country of Bangladesh, compared factors of healthy lifestyle practices (exercising, avoiding smoking), self-help (attending appointments, following treatment regimens), and knowledge and awareness about diabetes control among those individuals on insulin versus non-insulin (tablet, diet) treatment, and those with a longer diabetes duration (≥5 years) versus a shorter duration. The study [16] found a significantly greater percentage of participants on insulin (compared to those not on insulin) or with diabetic duration ≥5 years (compared to those with <5 years) self-reported not engaging in regular exercise, not adhering strictly to their treatment regimen, and were not aware of whether their diabetes was well controlled, despite being at an increased risk of developing retinopathy complication from uncontrolled diabetes. These findings led to the development of an effective patient-centered intervention program to improve awareness and lifestyle practices, in which unique challenges reported by participants with known higher risk of diabetes-related blindness (on insulin treatment and/or longer duration of diabetes) were addressed [28].

Although several previous studies from Bangladesh have examined the KAP parameters in people with and without diabetes [19, 29–31], there is a paucity of data comparing how diabetes KAP parameters differ between individuals on insulin versus non-insulin treatment, and other studies have not explored all the parameters explored in this study. This study aimed to compare diabetes-related KAP parameters between participant groups with different durations of diabetes (up to six years or longer than six years as the median duration of the participants was 6 years) and the type of treatment (insulin or insulin combined with tablet versus non-insulin (tablet and/or diet control)). Furthermore, the study examined how various sociodemographic factors such as age, gender, and educational level influence diabetes-related KAP parameters in high-risk groups for blindness due to uncontrolled diabetes, as very little research evidence exists on this for Bangladesh. Identifying the parameters significantly associated with knowledge about diabetes-related blindness would be useful for designing targeted educational interventions to improve patients’ attendance at diabetic retinopathy (DR) screening services and prevent diabetes-related blindness in Bangladesh. Evidence shows that the uptake of DR screening in Bangladesh is suboptimal [32].

Our study will also shed light on the challenges of self-management faced by people in Bangladesh who may have limited access to healthcare services due to factors such as a shortage of healthcare centers, remote locations, or unreliable transportation [33, 34].

## Materials and methods

### Participants

Six hundred individuals with diabetes (mean age = 52.7 ±11.6 years, range = 23-95 years) from the urban community of the Central-Western zone of Bangladesh were recruited using a purposive sample method. All participants provided informed consent to take part in the study. Participants were treated following applicable ethical guidelines that followed tenets of Helsinki Declaration. The Uttara Adhunik Medical College ethics review committee, Uttara, Dhaka-1230, Bangladesh [Ref. No.: UAMC/ERC/03/2022/12] approved the study protocol.

### Sample size calculation

The sample size was calculated using the following equation:

$$n=\frac{{z}^{2}pq}{{d}^{2}}; n=\frac{{1.96}^{2}\times 0.579\times \left(1-0.579\right)}{{0.05}^{2}}=374.57\approx 375$$

Here,

*n* = number of samples

*z* = 1.96 (95% confidence level)

*p *= prevalence estimate (57.9% or 0.579) (30)

*q* = (1-*p*)

*d* = margin of error (0.05)

A previous study conducted in Bangladesh among individuals with Type 2 Diabetes reported 42.1% poor knowledge and 57.9% fair/good/very good knowledge [30]. Based on the prevalence estimate, the minimum sample size was estimated as 375. However, the present sample was exceeded the minimum requirement which indicating the good power of study. Following the receipt of informed consent, 607 individuals were interviewed. 7 individuals with incomplete data were eliminated, leaving 600 in the final analysis.

### Procedures

Data were collected by a research assistant using a semi-structured questionnaire (Tables 1, 2, 3, 4 and 5) in the Bangladeshi language between July and September 2022 under the supervision of an established researcher. The questionnaire was validated and was similar to the one used in our previous studies from Nepal, China, and India [16, 28, 35]. The questionnaire collected information about demographics, duration, and treatment of diabetes, and diabetes-related KAP, including the number of times participants forgot to take their diabetes medication, hospital visits for uncontrolled diabetes, and lifestyle factors such as smoking, physical activity, and diet.

The questionnaire was translated into the local language and then back-translated into English, which a dual-language expert reviewed. All participants were able to respond to all the questions without any help. The questionnaire was pretested in 10 participants with diabetes who did not take part in the final study. All the queries arising from the pretesting were addressed before the final data collection was conducted.

Participants who were receiving insulin or insulin combined with a tablet for their diabetes treatment were categorized in the ‘*insulin*’ group, and those receiving tablet only and/or diet control treatment were categorized in the ‘*non-insulin*’ group. Similarly, participants with a diabetes duration longer than the overall median duration (in our case, 6 years) were categorized in the ‘*longer duration*’ group. Those with a shorter duration (up to six years) were categorized in the ‘*shorter duration*’ group. A similar categorization approach has been previously used elsewhere [16]. Data were analyzed using SPSS statistical software (version 29). Associations of demographic and diabetes-related clinical data with the type of diabetes treatment, duration of diabetes, and awareness about whether diabetes can cause blindness were explored using Fisher’s exact test. Independent-sample t-tests were used to analyze continuous variables such as age, number of episodes of uncontrolled diabetes, and the number of times forgotten to take diabetic medication in the previous month. Appropriate data reporting guidelines (Strengthening the Reporting of Observational Studies in Epidemiology, STROBE) were followed [36].

## Results

Table 1 provide the overall summary of data from 600 participants (mean age = 52.7 ±11.6 years).

We discuss the main findings from Table 1 below.

**Table 1:** Overall summary of demographics (A), diabetes-related information, and KAP data (B) from 600 participants

**A**			
**Demographics Variable**		**Category**	**No. of participants (%) (*n* = 600)**
Gender		Male	271(45.2%)
		Female	329(54.8%)
Education level		None	147(24.5%)
		≥primary	453(75.5%)
Employment status		Employed/self-employed	433(72.2%)
		Unemployed	167(27.8%)
Mean age in years (SD)			52.7±11.6
**B**			
**Variable**		**Category**	**No. of participants (%) (*n* = 600)**
Duration of diabetes		≤6 years	381(63.5%)
		>6 years	219(36.5%)
Treatment for diabetes		Non-insulin (Tablet, Diet)	116(19.3%)
		Insulin/insulin and tablet	484(80.7%)
Do you take medicine for high BP?		Yes	405(67.5%)
		No	195(32.5%)
Do you take medicine for cholesterol?		Yes	300(50%)
		No	300(50%)
**Knowledge:**			
Is BP control important for controlling diabetes?		Yes	420(70%)
		No/not sure	180(30%)
Is cholesterol control important for controlling diabetes?		Yes	345(57.5%)
		No/not sure	255(42.5%)
Is diabetes a serious disease?		Yes	481(80.2%)
		No/not sure	119(19.8%)
Is the responsibility for the control of diabetes yours, doctors, or both or someone else?		My doctor’s	8(1.3%)
		Mine	266(44.3%)
		Me & my doctor	320(53.3%)
		Someone else	6(1.0%)
Is exercise important to control diabetes?		Yes	578(96.3%)
		No/not sure	22(3.7%)
Is exercise important to control diabetes?		Yes	578(96.3%)
		No/not sure	22(3.7%)
Is smoking harmful for your diabetes?		Yes	361(60.2%)
		No/not sure	239(39.8%)
Can diabetes cause blindness?		Yes	412(68.7%)
		No/not sure	188(31.3%)
Are eye tests for spectacles and diabetes different?		Yes	235(39.2%)
		No/not sure	365(60.8%)
When should you get your eyes tested for diabetes?		Only when sight worsens	249(41.5%)
		Immediately after diagnosis	285(47.5%)
		One year after diagnosis	66(11.0%)
**Attitude:**			
Would you worry if you forgot to take your medication?	Yes		443(73.8%)
	No/not sure		157(26.2%)
Would you be comfortable telling people that you have diabetes?	Yes		313(52.2%)
	No/not sure		287(47.8%)
Would it be difficult to maintain a healthy diet during festivals?	Yes		489(81.5%)
	No/not sure		111(18.5%)
Would you go to get your eyes tested for diabetes if you are seeing well?	Yes		254(42.3%)
	No/not sure		346(57.7%)
**Practice:**			
Do you carry regular physical exercise?	Yes		521(86.8%)
	No/not sure		79(13.2%)
When did you last have your eyes checked?	Within the last one year		302(50.3%)
	Within the last 1-2 years		55(9.2%)
	Never		243(40.5%)
Do you smoke?	Yes		180(30%)
	No		420(70%)
How often do you check your blood sugar?	Once within a day-month		413(68.8%)
	Once within a month-year		179(29.8%)
	Don’t know		8(1.3%)
Have you told your family that you have got diabetes?	Yes		589(98.2%)
	No		11(1.8%)

There were 271 male (mean age = 54.4 years±12.8) and 329 female (mean age = 51.3 years±10.3) participants. A quarter (24.5%) of all participants reported not having any formal education, and 27.8% were unemployed.

Out of the total participants, 70% reported that blood pressure (BP) control was important in controlling diabetes, but only 57.5% reported that cholesterol control was important in controlling diabetes. Fifty percent of participants stated they were taking medication for high cholesterol and 67.5% for high BP.

One-fifth (19.8%) of all participants did not consider diabetes a serious disease. Nearly half of all participants (46.7%) reported not knowing that diabetes management required self-management as well as care offered by their doctor,

Although a high percentage of participants (96.3%) reported that physical activity was important for controlling diabetes, a lower percentage (86.8%) actually engaged in regular physical activities such as walking, running, or cycling, Thirty percent of all participants smoked and 39.8% did not know that smoking is harmful to diabetes control.

Of the total participants, 73.8% would be concerned if they forgot to take their diabetes medicine, and 68.8% said they check their blood sugar at least once a month.

Two-fifths (40.5%) of the participants reported never having had their eyes tested for diabetic retinopathy. Nearly one-third of the total participants (31.3%) did not know that uncontrolled diabetes can cause blindness. Furthermore, 41.5% of the total participants said that they would present for a diabetic eye test only when their eyesight worsens.

### Comparison of participants in the ‘insulin’ group (who were on insulin on its own or insulin combined with tablet treatment) and ‘non-insulin’ group (who were on tablet and/or diet control treatment)

Out of the total 600 participants, 80.7% participants were in the *insulin* group, and the remaining (19.3%) in the *non-insulin* group.

Table 2 examines the association of demographic and diabetes-related clinical data with the type of treatment (insulin/non-insulin). Table 3 examines how the exposure variable of the type of diabetes treatment (insulin/non-insulin) is associated with diabetes-related KAP parameters as the outcome variables.

We discuss key significant findings from Tables 2 and 3 below:

**Table 2:** Examining the association of demographic and diabetes-related clinical data with the type of diabetes treatment (insulin/non-insulin)

**Variable**	**Category**	**Treatment type**		***P*-value** (Fisher exact test)
		**Tablet/diet** **(*n* = 116)**	**Insulin/Insulin & tablet** **(*n* = 484)**	
Gender	Male	47.4%	44.6%	0.61
	Female	52.6%	55.4%	
Education level	No formal education	12.1%	27.5%	<0.001*
	≥primary	87.9%	77.5%	
Employment status	Employed/self-employed	65.5%	73.8%	0.08
	Unemployed	34.5%	26.2%	
Duration of diabetes	≤6 years	76.7%	60.3%	<0.001*
	>6 years	23.3%	39.7%	
Do you take medicine for high BP?	Yes	37.9%	74.6%	<0.001*
	No	62.1%	25.4%	
Do you take medicine for cholesterol?	Yes	21.6%	56.8%	<0.001*
	No	78.4%	43.2%	
Mean age (SD)		47.6±11.5	53.9±11.2	<0.001*

^*^represents significant *p*-values (*p*< 0.05)

**Table 3:** Type of diabetes treatment (insulin/non-insulin) as an exposure variable and diabetes-related KAP parameters as the outcome variables

**Variable**	**Category**	**Treatment type** **Tablet/diet** **(*n* = 116)**	**Insulin/Insulin & tablet** **(*n* = 484)**	***P*-value** (Fisher exact test)
Is BP control important for controlling diabetes?	Yes	77.6%	68.2%	0.05*
	No/not sure	22.4%	31.8%	
Is cholesterol control important for controlling diabetes?	Yes	69.8%	54.5%	0.003*
	No/not sure	30.2%	45.5%	
Is diabetes a serious disease?	Yes	78.4%	80.6%	0.61
	No/not sure	21.6%	19.4%	
Is the responsibility for the control of diabetes yours, doctors, both or someone else?	My doctor	0.9%	1.4%	0.32
	Mine	50%	43%	
	Mine & doctor	47.4%	54.8%	
	Someone else	1.7%	0.8%	
Would you worry if you forgot to take your medication?	Yes	58.6%	77.5%	<0.001*
	No/not sure	41.4%	22.5%	
Do you do regular physical exercise?	Yes	83.6%	87.6%	0.24
	No	14.7%	11.8%	
	Don’t know	1.7%	0.6%	
Have you told your family that you have got diabetes?	Yes	94%	99.2%	0.001*
	No	6%	0.8%	
Would you be comfortable telling people that you have diabetes?	Yes	50.9%	52.5%	0.76
	No/not sure	49.1%	47.5%	
Would it be difficult to maintain a healthy diet during festivals?	Yes	84.5%	80.8%	0.43
	N0/not sure	15.5%	19.2%	
Is exercise important to control diabetes?	Yes	94.8%	96.7%	0.41
	No/not sure	5.2%	3.3%	
Is smoking harmful for your diabetes?	Yes	69.8%	57.9%	0.02*
	No/not sure	30.2%	42.1%	
Are eye tests for spectacles and diabetes different?	Yes	55.2%	35.3%	<0.001*
	No/not sure	44.8%	64.7%	
Would you go to get your eyes tested for diabetes if you were seeing well?	Yes	44.8%	41.7%	0.6
	No/not sure	55.2%	58.3%	
When should you get your eyes tested for diabetes?	Only when sight worsens	44.8%	40.7%	0.94
	Immediately after diagnosis	41.4%	49%	
	One year after diagnosis	13.8%	10.3%	
When did you last have your eyes checked?	Within one year	54.2%	49.7%	0.54
	Within 1-2 years	6.5%	9.8%	
	Never	39.3%	40.6%	
Do you smoke?	Yes	30.2%	30%	0.99
	No	69.8%	70%	
How often do you check your blood sugar?	Once within a day-month	67.2%	69.2%	0.53
	Once within a month-year	30.2%	29.8%	
	Don’t know	2.6%	1%	
Mean no. of times forgotten to take tablet or insulin in the last month		2.3±3.3	2.1±2.3	0.66

^*^represents significant *p*-values (*p*< 0.05)

Participants in the insulin group were significantly older than in the non-insulin group (*p*< 0.001). The proportion of participants with no formal education was significantly higher in the insulin group when compared to the non-insulin group (27.5% and 12.1% respectively, *p*< 0.001). A significantly greater percentage of those in the insulin group (39.7%) had a longer duration of diabetes (>6 years) compared to those in non-insulin group (23.3%) (*p*< 0.001) and were on high BP and cholesterol medications (74.6% and 56.8%, respectively) when compared to non-insulin group (37.9% and 21.6% respectively). Despite this, a significantly greater proportion of participants in the insulin group were not aware that BP and cholesterol control are important for controlling diabetes (31.8% and 45.5%, respectively) when compared to those in the non-insulin group (22.4% and 30.2%, respectively).

A higher percentage of participants in the insulin group (42.1%) were not aware of the negative effects of smoking compared to those in the non-insulin group (30.2%).

More participants in the insulin group were unaware of the difference between eye tests for spectacles and diabetic-related eye changes (64.7%) compared to the non-insulin group (44.8%), suggesting a lack of knowledge and awareness about diabetes and its complications in the eye. On the other hand, 77.5% in the insulin group reported that they would be concerned if they forgot to take their diabetes medicine, compared to 58.6% in the non-insulin group.

### Longer than 6 years versus shorter duration of diabetes

We compared various parameters in participants who had diabetes duration longer than six years (*longer-duration* group) and up to 6 years (*shorter-duration* group). Six years was chosen as the cut-off off as the median duration of diabetes in the group was 6 years.

Out of the total 600 participants, 36.5% reported having a diabetic duration of longer than 6 years. Table 4 examines the association of demographic and diabetes-related clinical data with the duration of diabetes (up to six years and more than six years). Table 5 examines the association of the duration of diabetes as the exposure variable with diabetes-related KAP parameters as the outcome variables.

We discuss key significant findings from Tables 4 and 5 below.

**Table 4:** Duration of diabetes (up to six years versus more than six years) and demographic characteristics and diabetes-related clinical data of high BP and cholesterol

**Variable**	**Category**	**Duration of diabetes**		***P*-value** (Fisher exact test)
		**≤6 years (*n* = 381)**	**>6 years (*n* = 219)**	
Gender	Male	44.9%	45.7%	0.86
	Female	55.1%	54.3%	
Education level	No formal education	22.8%	27.4%	0.24
	≥primary	77.2%	72.6%	
Employment status	Employed/self-employed	76.4%	64.8%	0.002*
	Unemployed	23.6%	35.2%	
Treatment type	Diet only/tablet	23.4%	12.3%	<0.001*
	Insulin/combined	76.6%	87.7%	
Do you take medication for blood pressure?	Yes	59.1%	82.2%	<0.001*
	No	40.9%	17.8%	
Do you take medication for cholesterol?	Yes	43.6%	61.2%	<0.001*
	No	56.4%	38.8%	
Mean age (SD)		49.8±11.6	57.7±9.6	<0.001*

*represents significant *p*-values (*p*< 0.05)

**Table 5:** Duration of diabetes as an exposure variable and diabetes-related KAP parameters as the outcome variables

**Variable**	**Category**	**Duration of diabetes**		***P*-value** (Fisher exact test)
		**≤6 years (*n* = 381)**	**>6 years (*n* = 219)**	
Is blood pressure control important for controlling diabetes?	Yes	69.8%	70.3%	0.93
	No/not sure	30.2%	29.7%	
Is cholesterol control important for controlling diabetes?	Yes	59.3%	54.3%	0.26
	No/not sure	40.7%	45.7%	
Is diabetes a serious disease?	Yes	80.3%	79.9%	0.92
	No/not sure	19.7%	20.1%	
Is the responsibility for the control of diabetes yours, doctors, both or someone else?	My doctor	1.3%	1.4%	<0.001*
	Mine	49.9%	34.7%	
	Mine & doctor	48.3%	62.1%	
	Someone else	0.5%	1.8%	
Would you worry if you forgot to take your medication?	Yes	73.8%	74%	0.99
	No/not sure	26.2%	26%	
Do you do regular physical exercise?	Yes	87.9%	84.9%	0.7
	No	10.8%	15.1%	
	Don’t know	1.3%	0%	
Have you told your family that you have got diabetes?	Yes	97.6%	99.1%	0.34
	No	2.4%	0.9%	
Would you be comfortable telling people that you have got diabetes?	Yes	44.6%	65.3%	<0.001*
	No/not sure	55.4%	34.7%	
Would it be difficult to maintain a healthy diet during festivals?	Yes	83.7%	77.6%	0.08
	No/not sure	16.3%	22.4%	
Is exercise important to control diabetes?	Yes	96.6%	95.9%	0.82
	No/not sure	3.4%	4.1%	
Is smoking harmful for your diabetes?	Yes	57.5%	64.8%	0.08
	No/not sure	42.5%	35.2%	
Are eye tests for spectacles and diabetes different?	Yes	36%	44.7%	0.04*
	No/not sure	64%	55.3%	
Would you go to get your eyes tested for diabetes if you were seeing well?	Yes	39.9%	46.6%	0.12
	No/not sure	60.1%	53.4%	
When should you get your eyes tested for diabetes?	Only when sight worsens	43.6%	37.9%	0.13
	Immediately after diagnosis	47.2%	47.9%	
	One year after diagnosis	9.2%	14.2%	
When did you last have your eyes checked?	Within the last year	63.5%	21.3%	<0.001*
	Within the last 1-2 years	10.0%	7.4%	
	Never	26.5%	71.3%	
Do you smoke?	Yes	28.9%	32%	0.46
	No	71.1%	68%	
How often do you check your blood sugar?	Once within a day-month	69%	68.5%	0.87
	Once within a month-year	29.1%	31.1%	
	Don’t know	1.8%	0.4%	
Mean no. of times forgotten to take tablet or insulin in the last month		2.0±2.6	2.5±2.4	0.02*

*represents significant *p*-values (*p*< 0.05)

Participants in the longer-duration group were significantly older than those in the shorter-duration group (*p*< 0.001). A significantly greater proportion of participants with a diabetes duration longer than six years were on medication for high BP and cholesterol (82.2% and 61.2%, respectively) compared to those with a shorter duration (59.1% and 43.6%, respectively). More participants with a longer duration of diabetes reported being aware of the joint responsibility (theirs and the doctor’s) in managing their diabetes (62.1%) compared to the shorter duration (48.3%) group. Participants in the longer-duration group were also more comfortable about revealing their diabetes status (65.3%) compared to those in the shorter-duration group (44.6%).

A higher proportion of participants in the longer duration group were aware of the difference between eye tests for spectacles and diabetes-related eye disease (44.7%) compared to the shorter duration (36.0%) group. A higher percentage in the shorter duration group reported having had their eyes tested for diabetic changes within the previous year (63.5%) compared to those in the longer duration (> six years) group (21.3%). Participants in the longer duration group reported forgetting to take their diabetic medication in the previous month more times (2.5±2.4) compared to those in the shorter duration group (2.0±2.6) (p = 0.02).

Tables 6 provide comparisons of demographic data and diabetes-related clinical data between participant groups who were aware and not aware of whether diabetes can cause blindness. Table 7 examines how the exposure variable of awareness of whether diabetes can cause blindness is associated with the diabetes-related KAP parameters as the outcome variables.

We discuss the main findings from Tables 6 and 7, below.

**Table 6:** Awareness of whether diabetes can cause blindness and demographic characteristics and diabetes-related clinical data

**Categorical Variable**	**Category**	***Awareness* about whether diabetes can cause blindness**		***P*-value (**Fisher exact test)
		Yes (*n* = 412)	No/not sure (*n* = 188)	
Gender	Male	44.4%	46.8%	0.60
	Female	55.6%	53.2%	
Education level	None	22.1%	29.8%	0.05*
	≥primary	77.9%	70.2%	
Employment status	Employed/self-employed	71.1%	74.5%	0.43
	Unemployed	28.9%	25.5%	
Duration of diabetes	≤6 years	59.7%	71.8%	0.005*
	>6 years	40.3%	28.2%	
Treatment for diabetes	Non-insulin (Tablet, Diet)	18.7%	20.7%	058
	Insulin/insulin and tablet	81.3%	79.3%	
Do you take medicine for high BP?	Yes	76.2%	48.4%	<0.001*
	No	23.8%	51.6%	
Do you take medicine for cholesterol?	Yes	58.7%	30.9%	<0.001*
	No	41.3%	69.1%	
Mean age (SD)		53.6±11	50.7±12	0.005*

*represents significant *p*-values (*p*< 0.05)

**Table 7:** Awareness of whether diabetes can cause blindness as an exposure variable and diabetes-related KAP parameters as the outcome variables

**Categorical Variable**	**Category**	***Awareness* about whether diabetes can cause blindness**		***P*-value (**Fisher exact test)
		Yes (*n* = 412)	No/not sure (*n* = 188)	
Is BP control important for controlling diabetes?	Yes	73.8%	61.7%	0.004*
	No/not sure	26.2%	38.3%	
Is cholesterol control important for controlling diabetes?	Yes	62.4%	46.8%	<0.001*
	No/not sure	37.6%	53.2%	
Is diabetes a serious disease?	Yes	81.3%	77.7%	0.32
	No/not sure	18.7%	22.3%	
Is the responsibility for the control of diabetes yours, doctors, or both or someone else?	My doctor’s	0.5%	3.2%	<0.001*
	Mine	50.2%	31.4%	
	Me & my doctor	48.5%	63.8%	
	Someone else	0.7%	1.6%	
Would you worry if you forgot to take your medication?	Yes	71.8%	78.2%	0.11
	No/not sure	28.2%	21.8%	
Do you do regular physical exercise?	Yes	87.9%	84.6%	0.3
	No/not sure	12.1%	15.4%	
Have you told your family that you have got diabetes?	Yes	99.0%	96.3%	0.04*
	No	1.0%	3.7%	
Would you be comfortable telling people that you have diabetes?	Yes	54.9%	46.3%	0.05*
	No/not sure	45.1%	53.7%	
Would it be difficult to maintain a healthy diet during festivals?	Yes	84%	76.1%	0.02*
	No/not sure	16%	23.9%	
Is exercise important to control diabetes?	Yes	97.1%	94.7%	0.16
	No/not sure	2.9%	5.3%	
Is smoking harmful for your diabetes?	Yes	65.5%	48.2%	<0.001*
	No/not sure	34.5%	51.6%	
Are eye tests for spectacles and diabetes different?	Yes	48.1%	19.7%	<0.001*
	No/not sure	51.9%	80.3%	
Would you go to get your eyes tested for diabetes if you are seeing well?	Yes	54.1%	16.5%	<0.001*
	No/not sure	45.9%	83.5%	
When should you get your eyes tested for diabetes?	Only when sight worsens	28.9%	69.1%	<0.001*
	Immediately after diagnosis	56.3%	28.2%	
	One year after diagnosis	14.8%	2.7%	
When did you last have your eyes checked?	Within the last one year	63.6%	21.3%	**<0.001**
	Within the last 1-2 years	10%	7.4%	
	Never	26.4%	71.3%	
Do you smoke?	Yes	32.3%	25%	0.08
	No	67.7%	75%	
How often do you check your blood sugar?	Once within a day-month	70.4%	65.4%	0.02*
	Once within a month-year	29.1%	31.4%	
	Don’t know	0.5%	3.2%	
Mean no. of times forgotten to take tablet or insulin in the last month		2.4±2.7	1.5±2.1	<0.001*

^*^represents significant *p*-values (*p*< 0.05)

A higher percentage of participants who were aware that uncontrolled diabetes may cause blindness had an eye check within the previous year compared to those who were not aware (*p*< 0.001). Those who reported to getting their blood sugar checked at least once a month were also more aware that uncontrolled diabetes may cause blindness (*p*= 0.02). On the other hand, participants who were aware that uncontrolled diabetes may cause blindness forgot to take their diabetes medicine more often compared to those who were not aware (*p* <0.001).

A significantly greater proportion (54.1%) of those who were aware that uncontrolled diabetes may cause blindness reported to having their eyes checked even if they were seeing well compared to those who were not aware (16.5%). Also, a significantly greater proportion of those who were aware that uncontrolled diabetes may cause blindness reported that it would be difficult to maintain a healthy diet during festivals (84.0%) compared to those who were not aware (76.1%).

## Discussion

This is the first study to examine the different participant groups categorized according to their treatment type or duration of diabetes. In this community-based study conducted in an urban setting of Central-Western Bangladesh, we explored associations between the duration of diabetes (longer and shorter than six years) and treatment type (on insulin or not) with sociodemographic factors including age, gender, educational level as well as their knowledge/awareness, attitude, and self-help and lifestyle practices (e.g., smoking, exercising). To the best of our knowledge, this is the first study to directly compare the diabetes-related KAP among diabetic individuals categorized by treatment type and the duration of diabetes, focusing on those who are at higher of risk of blindness [27]. Our findings offer significant evidence to inform the design of targeted interventions to prevent blindness from uncontrolled diabetes in these high-risk groups in Bangladesh.

While a substantial proportion of individuals self-reported the importance of BP and cholesterol control, physical exercise, negative effects of smoking, and the importance of discussing their diabetes with family members for support, several important factors need to be addressed through improved KAP, healthcare access, and policies. For example, nearly one-fifth (19.8%) of all the participants did not consider diabetes as a serious disease. A considerable number (40.5%) had never had their eyes tested for diabetes-related changes and a significant percentage (31.3%) reported not being aware that diabetes may cause blindness. The lack of *awareness* was more apparent in those with no formal education.

Our community-based study complements a hospital-based study from Dhaka (the capital city of Bangladesh) which reported a comparatively higher percentage (63%) of people who were not aware that diabetes could cause blindness [37], as well as another population-based study in which nearly 40% of individuals with diabetes over the age of 40 years were not aware that blindness due to diabetes could be prevented [32]. Improving eye health awareness would be vital in preventing vision loss due to diabetes [32, 38].

A substantial percentage (46.7%) of our participants reported not being aware that diabetes management is a shared responsibility between themselves and their healthcare providers, suggesting a need for improved communication between patients and healthcare providers. Healthy lifestyle practices such as reduced smoking behavior and increased physical activity are also to be encouraged, as a significant proportion (39.8%) were not aware that smoking is harmful to diabetes, with nearly one-third (30%) reporting smoking cigarettes. Interestingly, 68.8% of our participants reported checking their blood glucose levels at least once a month, whilst a previous study from the rural setting of Bangladesh reported a much lower percentage (22%) [22], suggesting different exposures to awareness and healthcare access in different parts of Bangladesh.

When comparing different treatment groups, a higher percentage of individuals in the insulin group were also on high BP (74.6%) and cholesterol (56.8%) medications, indicating a higher prevalence of comorbidities [39] or an advanced stage of diabetes requiring mixed (insulin and tablet) treatment [40]. A significant lack of awareness among individuals in the insulin group with regards to the importance of BP and cholesterol control highlights the need for targeted education interventions. A significantly higher proportion of participants in the insulin group were less aware of the harmful effects of smoking, emphasizing the need for smoking cessation education programs. The percentage of individuals with no formal education was also significantly higher in the insulin group.

Being aware that uncontrolled diabetes may lead to blindness was significantly associated with higher attendance for eye tests and also of regular monitoring of blood sugar. However, a significantly higher proportion of those who were aware that uncontrolled diabetes may cause blindness missed their medication more often as did participants in the longer duration group, indicating a need for sustained awareness programs on the need to ensure timely compliance with their treatments.

To summarize, our study provides evidence that people with diabetes in community settings in Bangladesh lack adequate knowledge and awareness about diabetes and its control, and engage less in self-care practices, with notable differences between those groups who have a longer duration of diabetes and are on insulin treatments. Addressing these issues requires targeted educational interventions to improve awareness about diabetes and its complications and promote self-care practices.

Our study has some limitations, including the inability to establish causality due to its cross-sectional nature. Additionally, as a community-based study, we did not have access to clinical measurements such as blood glucose, BP, and cholesterol to verify participants’ self-reported information. Nonetheless, our study provides important data to suggest that interventions of diabetes control should focus on at-risk groups for diabetes-related blindness including those on insulin, those with longer diabetes duration, and those with lower education levels. Addressing specific gaps in knowledge and self-care practices of diabetes is crucial for preventing complications such as diabetes-related blindness.

## Data Availability

The data generated may be obtained upon reasonable request to the corresponding author.
